# A Report of Two Cases from Notes Found in My Record Book, with Remarks

**Published:** 1895-05

**Authors:** T. Emerson Mitchell

**Affiliations:** Columbus, Ga.


### A REPORT OF TWO CASES FROM NOTES FOUND IN MY RECORD BOOK, WITH REMARKS.

By T. EMERSON MITCHELL, M.D.,

Columbus, Ga.

Case 1.—Miss A. McG., aged nineteen, came October 23, 1883, for consultation and advice as regards a double vision which had been a source of much annoyance to her since childhood, particularly when any form of near work was done, such as reading, embroidering or needle-work of any kind, the asthenopia becoming so intolerable at times as to necessitate laying aside all eye-work or covering one eye entirely, thereby obliterating one image. Upon examination it was found that with a plain red glass in front of either eye, the patient being seated twenty feet from a gas jet or candle flame, a crossed or heteronymous diplopia was manifest, a diffusion of the images only taking place when prismatic lenses, bases in, equal in strength to thirteen degrees were used. With the accommodation fully suspended (a condition that should never exist during an examination for heterophoria, fully explained by Savage in an article on the "Relationship between the Centers of Accommodation and Convergence," *Ophthalmic Record*, Vol. 2, No. 11, page 429) O. D. V $= \frac{20}{\text{XL}}$ w + 1.50 D. sphere and O.S.V. $= \frac{20}{\text{XV}}$ w + 1.00 D. sphere. P. D. $2\frac{6}{16}$, S. B. $\frac{12}{16}'' \times \frac{3}{16}''$ in plane. Gave for constant use O. D. + 1.00 D. sphere and O. S. + .50 D. sphere, and advised partial tenotomy of externi, which was for the present declined. With the hypermetropia thus partially corrected there was a diminution in the nervous impulse heretofore sent to the ciliary muscle to procure an acute image, and a corresponding diminution in the impulse sent to the already too weak interni caused by the disturbance of relation between the centers of convergence and accommodation incident to wearing the plus lenses.

As a result of this condition of things the hyperdeveloped externi asserted and maintained the mastery over their antagonistic lateral muscles, and, as a consequence, crossed diplopia existed at all times. Binocular fixation being impossible, on November 13, 1893, under cocaine anesthesia, I did a partial or graduated tenotomy of the right externus; but on testing for results afterwards, I found that there was still a marked diplopia; consequently the patient was again placed on the operating chair and the tendon completely severed from its scleral insertion, care being taken to leave all fascial or vaginal attachments intact.

A second examination for results showed an over-correction of six degrees, and notwithstanding the apparent indication for an immediate operation for advancing the severed tendon, I watched the effect daily diminish till December 1, 1893, when there was again a crossed diplopia of three degrees.

On March 19, 1894, finding a heteronymous diplopia with the red glass, I did a complete tenotomy of the left externus, scrupulously observing all the precautionary measures as to vaginal or fascial attachments as in the former instance, with a similar result of an over-correction of five degrees. This over-correction gradually subsided till June 9, 1894, when an examination showed binocular fixation, lateral orthophoria, and perfect freedom from eye symptoms, which comfort has continued to this writing, April 20, 1895; the lenses first prescribed being worn all the while.

I think this case teaches that in high degrees of exophoria, particularly if there is diplopia with a plane red glass in front of one eye, we should not be unduly solicitous about final results, if in doing a tenotomy an over-correction of a few degrees is produced. This, however, would not obtain in tenotomizing the interni, since after reattachment the effect is seldom less, but more frequently greater than is immediately obtained.

CASE 2.—Mr. J. T. W., aged twenty-four, came January 22, 1894, "to get a pair of glasses" to correct an imperfect vision which had developed and gradually grown worse during the three or four weeks preceding his visit to my office. Gave a specific history dating back seven years treated by a competent physician, apparent recovery. Patient said his general health was good, he ate well, slept well, felt well, and only wanted "a pair of glasses" that would enable him to see to resume his usual vocation—iron moulding. While his general appearance was fairly good, he had that tell-tale dejected facial expression and peculiar swarthy, almost indescribable pumpkin-like complexion that I have sometimes thought pathognomonic of Bright's disease. Upon a close examination he admitted that the renal secretion was possibly somewhat excessive in amount and that he had noticed some puffiness about the eyes more pronounced on rising in the morning. O. D. V. = $\frac{20}{CLX}$, O. S. V. = $\frac{20}{LX}$, not improved by any combination of lenses. An ophthalmoscopic view of the fundus oculi disclosed the swollen papillæ with imperfectly outlined edges, while distinct white spots or patches were seen about, or radiating from, the macula lutea, particularly noticeable in the right eye. Microscopically the urine was clear and limpid, not very unlike that of a diabetic patient, and contained many flakes or shreds of epithelium supposed to have been exfoliated from the mucous membrane of the bladder; specific gravity 1010 and loaded with albumin, fully 33⅓ per cent. by measure. Diagnosed albuminuric retinitis, and after explaining (to his friends) the gravity of the situation, advised the patient to go under the treatment of his family physician in conjunction with a "specialist" if he so desired.

My opinion was not acted upon, and the patient consulted an eminent ophthalmologist in an adjoining city, who confirmed my diagnosis and referred him to his home physician for treatment. It is sufficient to say that he rapidly grew worse till March 17th, less than sixty days from the time I first saw him, when the messenger came to relieve him of further earthly suffering. This shows how very insidiously Bright's disease may fasten itself to the very vitals of man, and how careful physicians should be not to ascribe every form of recent defective vision in which no pathological lesion is externally manifest, to some remote cause as "biliousness" or "malaria," on whose broad shoulders must rest the responsibility of a multitude of sins in the form of aches and pains. This leads me to say that impaired vision coincident with gestation is often treated too much as a matter of natural consequence by many practitioners and left to be righted by nature at the proper time, when, in reality, albuminuric retinitis is the immediate cause of this loss of function and demands radical interference at the hands of the obstetrician. In every case of defective ocular function during gestation the renal secretion should be repeatedly examined (at least once a week)

for albumin, and whenever this is found, particularly if the impaired vision be progressive and accompanied by œdema, and an occasional convulsion, artificial means for terminating the pregnancy should be resorted to. I have seen a patient who gave a history of impaired vision, anasarca, and repeated convulsive seizures, who was allowed to go on to full term, notwithstanding the mandatory indications for surgical interference, with the deplorable result of shutting the poor woman up for the rest of her life in darkness, so far as useful vision is concerned.

---

### TRIONAL AND TETRONAL.

In an exhaustive article, Egasse (*Bull. Gener. de Therapeutique*) reviews the literature regarding the therapeutic uses of these two drugs. The author, after an intelligent examination of the subject, is led to adopt the following conclusions: Trion and tetronal are not very toxic substances. Clinical trials show that trional especially is an excellent hypnotic, causing, particularly in the insane, a quiet sleep, its reparative influence being of long duration. It has the advantage over sulphonal in being rapid in action, even in small doses. Its bad after-effects are rare, and when they occur are much less marked than in the case of sulphonal. Trional is not an analgesic in the true sense of the term, but in many cases it acts as an hypnotic by diminishing pain. It may be associated with opium or morphine. The dose of trional should not exceed 45 or 60 grains. The average quantity to be administered may vary from 15 to 22½ grains, taking into consideration not only the age and sex of the individual, but also the character of the affection to be combated. Dosage, however, should be left to the judgment of the clinician. Trional may be given in cachets or capsules, or, better still, suspended in a warm mixture, which facilitates its solution and absorption. It ought to be administered half an hour before retiring at night. In this manner patients will not feel the vertigo which trional sometimes produces. This drug is, on the whole, an excellent hypnotic, destined to replace sulphonal.—*Therapeutic Gazette.*

